# Preclinical Immunogenicity Evaluation of a DTacP-sIPV/Hib Combination Vaccine in Rodent Models Under Varying Formulations and Immunization Schedules

**DOI:** 10.3390/vaccines13100993

**Published:** 2025-09-23

**Authors:** Yixian Fu, Wei Huang, Lukui Cai, Yan Ma, Qin Gu, Qiuyan Ji, Jingyan Li, Na Gao, Xiaoyu Wang, Guang Ji, Jiana Wen, Wenzhu Hu, Hongwei Liao, Ling Ping, Yuting Fu, Guoyang Liao, Lujie Yang, Shengjie Ouyang, Mingqing Wang, Xiaoyue He, Han Chu, Wenlu Kong, Xinhua Qin, Huimei Zheng, Jiangli Liang, Ting Zhao, Jingsi Yang

**Affiliations:** 1 Institute of Medical Biology, Chinese Academy of Medical Sciences & Peking Union Medical College, Kunming 650118, China; fyx0421@163.com (Y.F.);; 2 State Key Laboratory of Respiratory Health and Multimorbidity, Beijing 100005, China; 3 School of Life Sciences, Yunnan University, Kunming 650091, China

**Keywords:** DTacP-sIPV/Hib combination vaccine, Sabin IPV, Preclinical study

## Abstract

Background: Combination vaccines protecting against diphtheria, tetanus, pertussis, poliomyelitis, and Haemophilus influenzae type b reduce injection burden and improve compliance. While widely used globally, no domestically produced pentavalent vaccine is currently licensed in China. Recent updates to China’s immunization schedule—including earlier initiation and an added booster for pertussis—highlight the need for compatible combination vaccines. This study evaluated the immunogenicity and feasibility of a novel DTacP-sIPV/Hib candidate vaccine in preclinical models. Methods: The vaccine was assessed in NIH mice and Wistar rats. Two Hib dosages were tested in mice alongside a DTacP-wIPV/Hib vaccine (Pentaxim^®^). In rats, two sIPV formulations (Formulations A and B) were administered using different intervals (1-month vs. 2-month) and injection methods (mixed vs. separate). Antibody titers were measured by ELISA and poliovirus neutralization assays. Results: The candidate vaccine elicited robust immune responses in both models. In mice, after three doses, the high-dose Hib group achieved >90% seroconversion for pertactin antigen, whereas the low-dose group reached 100% for all antigens. In rats, antibody responses after three doses were comparable to those induced by Pentaxim^®^, with no significant differences between immunization schedules or administration routes. Compared with Formulation A (containing a higher type I sIPV antigen content), Formulation B exhibited reduced type I poliovirus neutralization after the first dose (*p* < 0.05) and delayed seroconversion, while responses to other antigens remained similar. Conclusion: The candidate DTacP-sIPV/Hib vaccine showed robust immunogenicity and flexibility across schedules and administration methods. A formulation including DT 12.5 Lf, TT 3.5 Lf, PT 25 μg, FHA 25 μg, PRN 8 μg, PRP 10 μg, and sIPV I/II/III at 30/32/45 DU is proposed for further development.

## 1. Introduction

Since the 1990s, diphtheria, tetanus, and acellular pertussis vaccines (DTaP), combined with inactivated poliovirus (IPV), Haemophilus influenzae type b (Hib), and hepatitis B (HBV) vaccines, have contributed to the development of quadrivalent, pentavalent, and hexavalent vaccines [1,2], which have been integrated into childhood immunization schedules in many developed countries [3]. However, no domestic pentavalent vaccine is currently available in China, and there are no pentavalent or higher valent combination vaccines included in the national immunization program now [4].

The global use of DTaP vaccines has greatly reduced disease incidence. However, pertussis remains a public health concern, with periodic resurgences observed in countries with high acellular pertussis (aP) vaccine coverage since the 1990s [5,6,7,8]. In China, 41,124 cases were reported in 2023 (2.92 per 100,000 population), rising sharply to over 470,000 cases in 2024 (34 per 100,000) [9,10]. China’s immunization program currently relies on co-purified aP vaccines. In contrast, acellular component pertussis (acP) vaccines purify each antigen separately, enabling precise formulation and potentially improved safety [11,12]. For the poliovirus component, most licensed combination vaccines worldwide use the wild-type Salk strain IPV (wIPV), which requires stringent biosafety conditions due to residual infectivity [13]. Sabin strain IPV (sIPV) offers a safer and more accessible alternative, and the WHO recommends its broader adoption to enhance global IPV supply [14,15,16].

Beginning January 2025, China revised its pertussis immunization schedule from doses at 3, 4, and 5 months to a 2, 4, and 6 month regimen, followed by boosters at 18 months and 6 years [17]. This transition highlights the need to evaluate candidate vaccines under both schedules, as evidence under the new program remains scarce.

The DTacP-sIPV/Hib combination vaccine candidate evaluated here was developed by the Institute of Medical Biology, Chinese Academy of Medical Sciences (IMBCAMS). It consists of a liquid DTacP-sIPV suspension and a lyophilized Hib component, reconstituted immediately prior to injection, consistent with the packaging of Pentaxim^®^, the only DTaP-wIPV/Hib vaccine currently marketed in China [18].

Therefore, this study aimed to assess the immunogenicity and preliminary safety of a novel DTacP-sIPV/Hib candidate vaccine in rodent models. Specifically, based on prior research on the DTacP-sIPV combination vaccine [19], the objectives were: (1) to determine the optimal Hib dosage using the NIH mouse model; (2) to evaluate two formulations of sIPV, corresponding to the currently licensed sIPV products in China, in Wistar rats to identify the optimal composition; (3) to compare immunogenicity under both the previous and the updated National Immunization Program schedules in China; (4) to investigate whether mixed versus separate administration of the DTaP-sIPV and Hib components affects vaccine immunogenicity in rats; and (5) to compare the immunogenicity of the candidate vaccine with that of Pentaxim^®^, the only licensed DTacP-wIPV/Hib vaccine in China. Furthermore, this study was designed to inform subsequent preclinical evaluation in non-human primates and to provide a scientific basis for defining the optimal formulation and immunization strategy for future clinical trials.

## 2. Materials and Methods

### 2.1. Animals

One-month-old NIH mice (10–12 g) and one-month-old Wistar rats (180–220 g) were obtained from the Experimental Animal Department at IMBCAMS (Animal Production License No. SCXK [Dian] K2022-0002) and housed under specific pathogen-free (SPF) conditions (License No. SYXK [Dian] K2022-0006). All animal procedures were approved by the Institutional Animal Care and Use Committee of IMBCAMS (Approval No. DWSP202110012) and conducted in SPF barrier environments to ensure animal health and experimental consistency.

During the acclimatization period, animals were identified using cage cards and sequentially numbered upon receipt, with males and females labeled separately (e.g., M001–M010, F001–F010). Animals were stratified by sex and body weight, then randomly assigned to the designated experimental groups using simple randomization. Allocation was performed by randomly selecting animals within each sex until groups reached the required sample size. The sample size was determined based on previous studies and empirical experience in similar vaccine immunogenicity models, while also taking into account ethical and practical considerations to minimize animal use. After grouping, detailed information including group number and animal ID was recorded on the cage cards to ensure accurate identification throughout the study. This procedure ensured that group allocation was unbiased and independent of animal order or housing. Mice received intraperitoneal injections of one-fifth the human vaccine dose, while rats were administered full human doses via intramuscular injection in the hind limbs. Blood samples were collected via the tail vein under aseptic conditions following proper disinfection. Routine procedures such as injection, blood collection, and weighing were performed without anesthesia to avoid potential interference with immune responses or sample quality. All operations were conducted by trained personnel to minimize animal stress and ensure welfare.

At the end of the experiment, mice were euthanized by cervical dislocation in accordance with institutional and international ethical guidelines for the humane treatment of laboratory animals. Rats were not euthanized during the study due to the long-term nature of immunogenicity assessments. However, if any animal exhibited severe health issues (e.g., visible or palpable large tumors), humane euthanasia was performed using an overdose of sodium pentobarbital via intravenous injection, following ethical guidelines.

### 2.2. Vaccine Preparation

The pentavalent diphtheria–tetanus–acellular component pertussis–Sabin inactivated poliovirus/Haemophilus influenzae type b (DTacP-sIPV/Hib) vaccine was developed under Good Manufacturing Practice (GMP) conditions. Each antigenic component was prepared separately prior to formulation. Corynebacterium diphtheriae and Clostridium tetani were cultured, and their toxins were extracted, purified, and detoxified to generate diphtheria toxoid (DT) and tetanus toxoid (TT). Bordetella pertussis was fermented, and the major antigens—pertussis toxin (PT), filamentous hemagglutinin (FHA), and pertactin (PRN)—were purified and detoxified before adsorption onto aluminum hydroxide adjuvant. For the poliovirus component, Sabin strains of type I, II, and III were propagated in Vero cells grown on microcarriers. The harvested viruses were purified, inactivated, and formulated into monovalent bulks, which were then blended proportionally to yield the trivalent Sabin inactivated poliovirus vaccine (sIPV). The Haemophilus influenzae type b (Hib) conjugate vaccine was produced by extracting and purifying the capsular polysaccharide (polyribosylribitol phosphate, PRP) from cultured bacteria and conjugating it to a carrier protein. The polysaccharide–protein conjugate was uniformly mixed with a lyophilization stabilizer, aliquoted into vials, and subsequently lyophilized.The final DTacP-sIPV/Hib vaccine consisted of a liquid DTacP-sIPV suspension and a lyophilized Hib component, which was reconstituted immediately prior to administration. The packaging format was consistent with Pentaxim^®^ (the only licensed DTaP-wIPV/Hib vaccine in China), which was used as the positive control in this study.

### 2.3. Grouping and Vaccination

#### 2.3.1. Study on the Immunogenicity and Immune Persistence of DTacP-sIPV/Hib Combination Vaccine in NIH Mice

**Figure 1:**
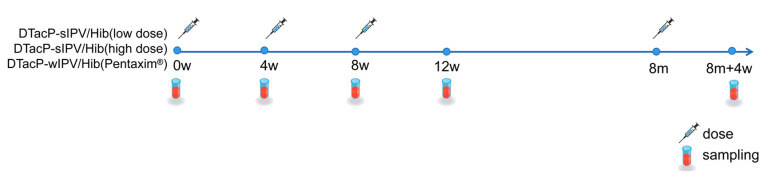
Immunization and blood collection schedule in NIH mouse.

24 SPF-grade NIH mouse were selected and randomly divided into three groups: the DTacP-sIPV/Hib low-dose group, the DTacP-sIPV/Hib high-dose group, and the positive control group, with eight mice per group. Vaccines for each group were formulated as described in Table 1. Intraperitoneal injections were administered to deliver the vaccines. The DTacP-sIPV/Hib low-dose group received a mixture of DTacP-sIPV and low-dose Hib, the DTacP-sIPV/Hib high-dose group received a mixture of DTacP-sIPV and high-dose Hib, and the positive control group received DTacP-wIPV/Hib vaccine Pentaxim^®^. The administered dose was one-fifth of the human dose, with a total injection volume of 0.5 mL per mouse. The primary immunization consisted of three doses, administered at 4-week intervals. A booster immunization (the 4th dose) was given 6 months after the third dose. The schedule of immunization and blood collection is illustrated in Figure 1. Since NIH mice are not considered an optimal model for evaluating poliovirus vaccines and are unsuitable for poliovirus neutralization assays, we proceeded with further evaluation in Wistar rats [20].

#### 2.3.2. Study on the Immunogenicity of DTacP-sIPV/Hib Combination Vaccine in Wistar Rats

A total of 110 SPF-grade Wistar rats were randomly allocated into 11 groups (10 rats per group) with equal sex distribution. Rats were individually identified by ear markings. Based on two currently approved sIPV vaccine formulations in China, the DTacP-sIPV/Hib groups were further subdivided into: DTacP-sIPV/Hib formulation A (containing IPV I: IPV II: IPV III = 30DU:32DU:45DU), DTacP-sIPV/Hib formulation B (containing IPV I: IPV II: IPV III = 15DU:45DU:45DU), positive control groups (Pentaxim^®^), and a blank control group (normal saline injection). Details of vaccine formulations are presented in Table 2.

**Figure 2:**
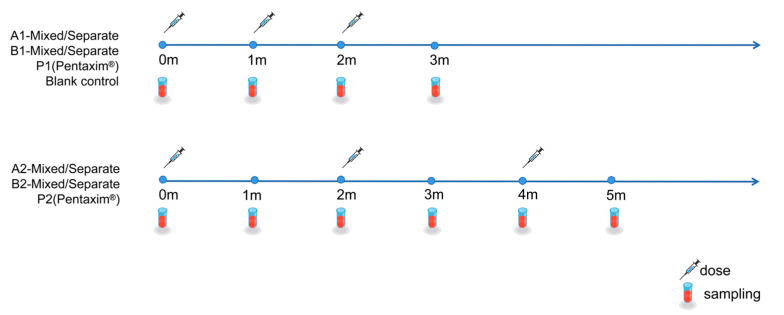
Immunization and blood collection schedule in Wistar rats.

Two administration methods were used: first, DTacP-sIPV/Hib was mixed before administration (0.25 mL of DTacP-sIPV/Hib was injected into each of the rat’s hind limbs); in the second, DTacP-sIPV and Hib were administered as separate injections (0.5 mL DTacP-sIPV in the left hind limb and 0.5 mL Hib, dissolved in saline, in the right). To accommodate the transition in the pertussis vaccine immunization schedule within China’s childhood immunization program, two distinct immunization schedules were designed for each vaccine formulation and administration method: one with 1-month intervals and another with 2-month intervals between vaccinations. For DTacP-sIPV/Hib formulation A, rats were categorized into four groups based on immunization intervals (1-month or 2-month) and administration methods (mixed injection of DTacP-sIPV and Hib, or separate administration), designated as A1-Mixed, A1-Separate, A2-Mixed, and A2-Separate groups. Similarly, DTacP-sIPV/Hib formulation B was divided into B1-Mixed, B1-Separate, B2-Mixed, and B2-Separate groups. The positive control groups received Sanofi Pasteur’s Pentaxim^®^ administered according to the manufacturer’s instructions, with P1 group following the 1-month interval schedule and P2 group the 2-month interval schedule. The blank control group received saline injections. To assess the safety of the candidate DTacP-sIPV/Hib vaccine during the primary immunization phase, body weight measurements were conducted in Wistar rats prior to each dose and on days 3 and 7 post-immunization. Additionally, body weight was recorded again 14 months after the first dose as long-term safety monitoring. Detailed group information is provided in Table 3. Blood samples were collected from the tail vein before immunization and 28 days post-vaccination. The immunization and blood collection schedule is summarized in Figure 2.

### 2.4. Serological Assays

Serum IgG antibodies against diphtheria toxoid (DT), tetanus toxoid (TT), filamentous hemagglutinin (FHA), pertactin (PRN), pertussis toxin (PT), and Haemophilus influenzae type b polyribosylribitol phosphate (PRP) were quantified by enzyme-linked immunosorbent assay (ELISA). For PT, FHA, and PRN in mouse sera, antibody concentrations (IU/mL) were calculated from standard curves generated by linear regression of optical density (OD_450_) values of reference standards. For DT, TT, and PRP in mouse sera, and for all six antigens (DT, TT, PT, FHA, PRN, PRP) in rat sera, titers were considered positive if they exceeded 2.1-fold the mean OD_450_ of blank wells. Thresholds for seropositivity were defined as follows: in mice, DT or TT titers ≥ 800, PRP titers ≥ 20, and PT, FHA, or PRN concentrations ≥ 20 IU/mL; in rats, DT, TT, PT, FHA, or PRN titers ≥ 800 and PRP titers ≥ 200. Neutralizing antibody titers against poliovirus types I, II, and III were determined using WHO-recommended virus neutralization assays [21], with titers ≥ 8 regarded as seropositive. Seroconversion was defined as the proportion of animals meeting these criteria post-immunization.

### 2.5. Statistical Analysis

Statistical analyses and data visualization were performed using GraphPad Prism (version 10.1.2). For the evaluation of geometric mean titers (GMTs) of antibodies, the Kruskal–Wallis H test was used to compare multiple groups at each time point because the data did not conform to a normal distribution, as assessed by the Shapiro-Wilk test. Dunn’s multiple comparisons test was applied for post hoc pairwise comparisons following the Kruskal–Wallis test to control the Type I error rate associated with multiple comparisons. For the assessment of Wistar rat body weight dynamics over time, repeated-measures two-way analysis of variance (two-way ANOVA) was conducted, with experimental group and time as the two factors. Body weights were measured at 10 time points spanning from before the first immunization to 14 months after the initial dose. The interaction between group and time was evaluated. All tests were two-tailed, and statistical significance was defined as *p* < 0.05. Actual *p*-values are reported in the text. Antibody titer data were visualized as bar graphs showing the median with interquartile range (IQR) to appropriately represent the non-normally distributed data.

## 3. Results

### 3.1. Study on the Immunogenicity and Immune Persistence of DTacP-sIPV/Hib Combination Vaccine in NIH Mice

#### Candidate DTacP-sIPV/Hib Vaccine Demonstrates Robust Immune Persistence in NIH Mice with Booster-Dependent PRN Antibody Recovery

**Figure 3:**
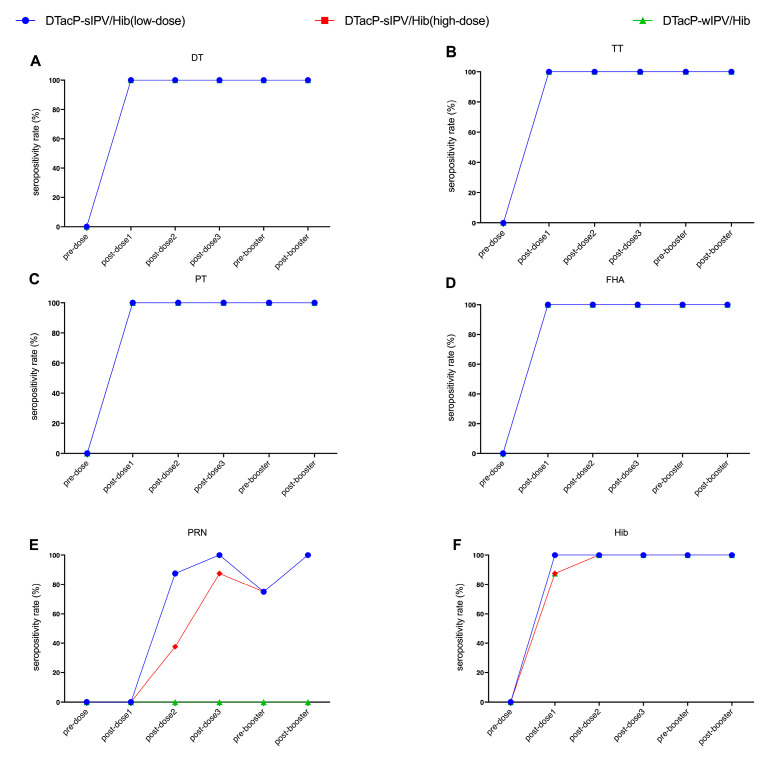
Seropositivity rates for DT (**A**), TT (**B**), PT (**C**), FHA (**D**), PRN (**E**), and Hib PRP (**F**) in NIH mice. DT (Diphtheria Toxoid); TT (Tetanus Toxoid); PT (Pertussis Toxin); FHA (Filamentous Hemagglutinin); PRN (Pertactin); PRP (Polyribosylribitol Phosphate).

In the DTacP-sIPV/Hib low-dose group, DTacP-sIPV/Hib high-dose group, and positive control DTacP-wIPV/Hib (Pentaxim^®^) group, the seroconversion rates for DT, TT, PT, and FHA antibodies were 100% at 28 days following the first dose and remained unchanged before and after the booster immunization (Figure 3). The seroconversion rates for PRP antibodies reached 100% at 28 days after the second immunization and also remained at 100% both before and after the booster immunization (Figure 3F). In the DTacP-sIPV/Hib low-dose group, the seropositivity rate for PRN antibodies reached 100% at 28 days after the third immunization, dropped to 75% at 6 months, and returned to 100% after the booster immunization (Figure 3E). In the DTacP-sIPV/Hib high-dose group, the seropositivity rate for PRN antibodies was only 87.5% at 28 days after the third immunization, dropped to 75% at six months, and reached 100% after the booster immunization. In the positive control DTacP-wIPV/Hib group, which lacks the PRN antigen, no PRN antibodies were detected.

**Figure 4:**
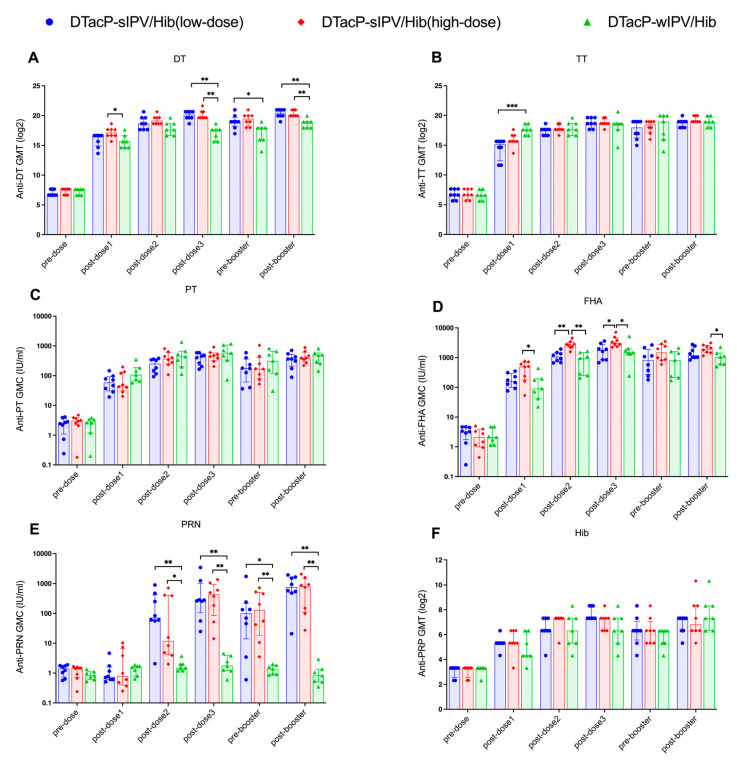
The GMTs of DT (**A**), TT (**B**), Hib PRP (**F**) antibodies and the GMCs of PT (**C**), FHA (**D**) and PRN (**E**) in mice were expressed as log2-transformed values ± 95% confidence intervals (CIs). Statistical significance was indicated as follows: * *p* < 0.05; ** *p* < 0.01; *** *p* < 0.001. Y axis for the GMCs of PT (**C**), FHA (**D**) and PRN (**E**) is shown on a logarithmic scale for better visualization of differences. DT (Diphtheria Toxoid); TT (Tetanus Toxoid); PT (Pertussis Toxin); FHA (Filamentous Hemagglutinin); PRN (Pertactin); PRP (Polyribosylribitol Phosphate); GMT (Geometric Mean Titer); GMC (Geometric Mean Concentration).

**Figure 5:**
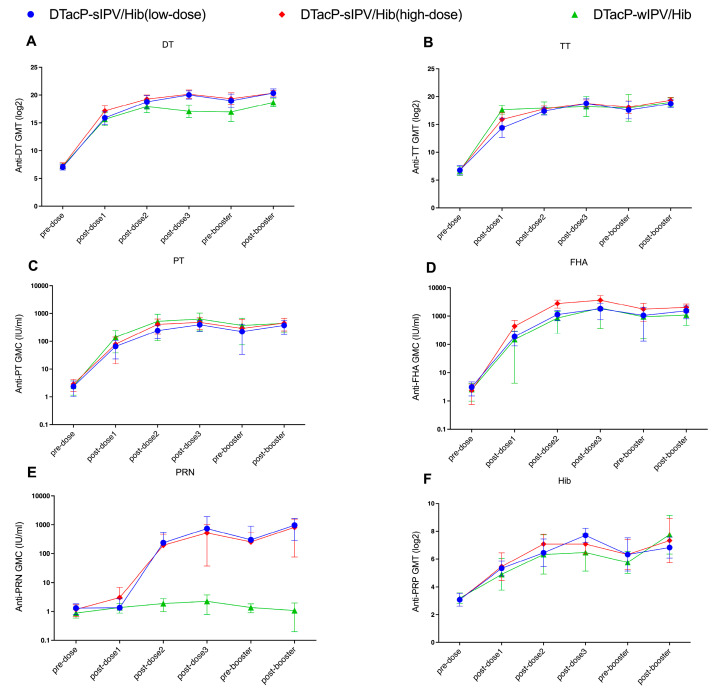
The trends of GMTs for DT (**A**), TT (**B**), and Hib PRP (**F**) antibodies and the GMCs of PT (**C**), FHA (**D**) and PRN (**E**) in mice, are presented as log2-transformed values ± 95% CIs. Y axis for the GMCs of PT (**C**), FHA (**D**) and PRN (**E**) is shown on a logarithmic scale for better visualization of differences. DT (Diphtheria Toxoid); TT (Tetanus Toxoid); PT (Pertussis Toxin); FHA (Filamentous Hemagglutinin); PRN (Pertactin); PRP (Polyribosylribitol Phosphate); GMT (Geometric Mean Titer); GMC (Geometric Mean Concentration).

Following the three-dose primary immunization series, DT antibody levels in both the low- and high-dose DTacP-sIPV/Hib groups were significantly higher than those in the DTacP-wIPV/Hib group (*p* = 0.0027 and *p* = 0.0022, respectively, Kruskal–Wallis H test) (Figure 4A). Prior to the booster immunization, DT levels in the low-dose group remained significantly higher than those in the DTacP-wIPV/Hib group (*p* = 0.0175, Kruskal–Wallis H test). After the booster immunization, DT levels in both the high- and low-dose groups were significantly higher than those in the DTacP-wIPV/Hib group (*p* = 0.0064 and *p* = 0.0077, respectively, Kruskal–Wallis H test). After the first immunization, TT antibody level in the DTacP-sIPV/Hib low-dose group was significantly lower than that in the DTacP-wIPV/Hib group (*p* = 0.0004, Kruskal–Wallis H test) (Figure 4B); however, no significant differences were observed at other time points. Compared to the DTacP-wIPV/Hib group, both DTacP-sIPV/Hib groups exhibited higher FHA antibody levels, although statistical significance was achieved only in the high-dose group (*p* = 0.0024 after the primary series; *p* = 0.0046 after the booster immunization, Kruskal–Wallis H test) (Figure 4D). As the DTacP-wIPV/Hib formulation does not contain the PRN component, the PRN antibody levels in both the DTacP-sIPV/Hib low-dose and high-dose groups were significantly higher than those in the DTacP-wIPV/Hib group after each immunization (Figure 4E). No significant differences were observed in PT or PRP antibody levels between the DTacP-sIPV/Hib low-dose and high-dose groups (Figure 4C,F). Figure 5 shows the temporal dynamics of antibody titers in mice. Except for FHA antibody levels after the second and third primary immunizations, which were significantly lower in the low-dose group compared to the high-dose group (*p* = 0.0067 and *p* = 0.0449, respectively, Kruskal–Wallis H test), no statistically significant differences in antibody levels for any component were observed between the two DTacP-sIPV/Hib groups at any time point (*p* > 0.05, Kruskal–Wallis H test).

### 3.2. Study on the Immunogenicity of DTacP-sIPV/Hib Combination Vaccine in Wistar Rats

#### 3.2.1. All Experimental Groups Achieved 100% Seroconversion Following Three-Dose Primary Immunization in Wistar Rats

**Figure 6:**
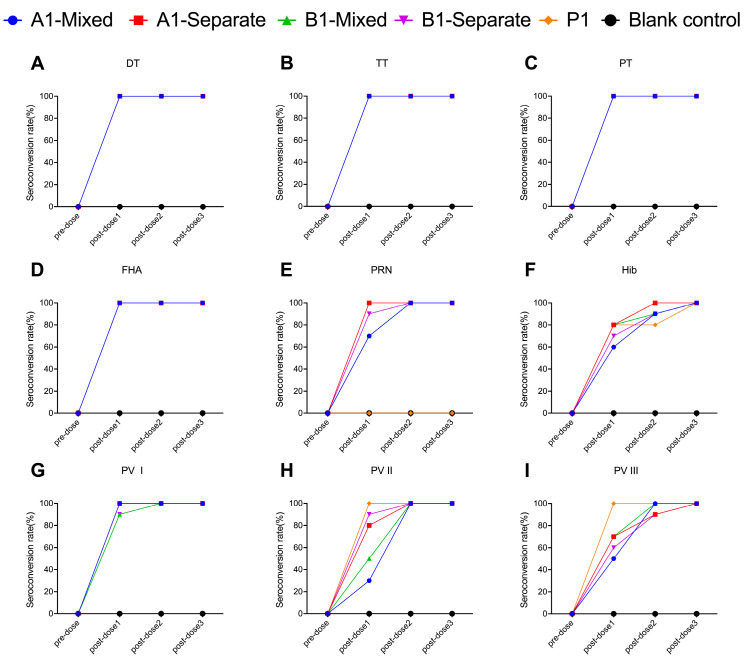
Seroconversion rates for DT (**A**), TT (**B**), PT (**C**), FHA (**D**), PRN (**E**), Hib PRP (**F**), and poliovirus types I (**G**), II (**H**), and III (**I**) in Wistar rats immunized with 1-month interval schedule. DT (Diphtheria Toxoid); TT (Tetanus Toxoid); PT (Pertussis Toxin); FHA (Filamentous Hemagglutinin); PRN (Pertactin); PRP (Polyribosylribitol Phosphate).

**Figure 7:**
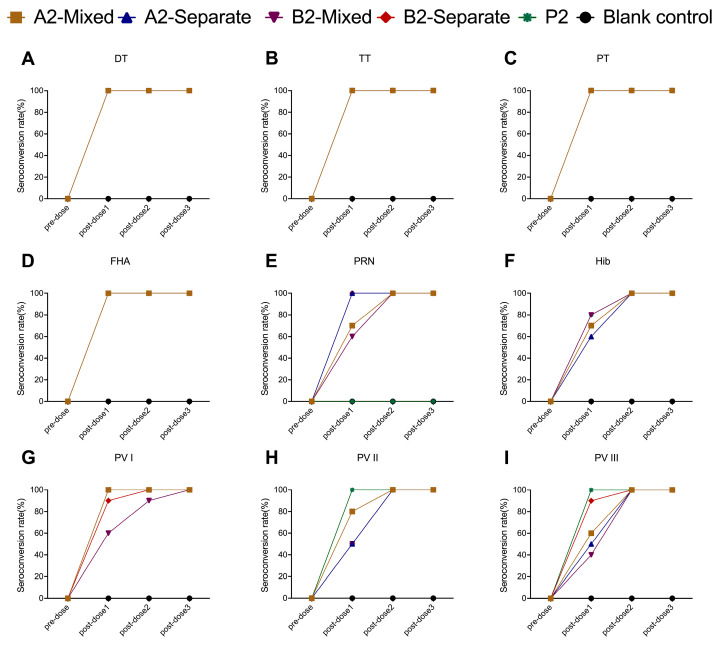
Seroconversion rates for DT (**A**), TT (**B**), PT (**C**), FHA (**D**), PRN (**E**), Hib PRP (**F**), and poliovirus types I (**G**), II (**H**), and III (**I**) in Wistar rats immunized with 2-month interval schedule. DT (Diphtheria Toxoid); TT (Tetanus Toxoid); PT (Pertussis Toxin); FHA (Filamentous Hemagglutinin); PRN (Pertactin); PRP (Polyribosylribitol Phosphate).

In all the experimental groups and the positive control groups (P1 group, P2 group), the seroconversion rates for DT, TT, PT, and FHA antibodies reached 100% at 28 days after the first immunization. In the experimental groups, the seroconversion rates for PRN antibodies reached 100% at 28 days after the second immunization. The positive control group received Pentaxim^®^, which lacks the PRN antigen and therefore does not induce PRN antibodies (Figure 6E and Figure 7E). Except for the A1-separate group, the seroconversion rates for PRP antibodies reached 100% at 28 days after the third immunization in the groups following the 1-month interval schedule (A1-mixed group, A1-separate group, B1-mixed group, B1-separate group, and P1 group) (Figure 6F). For groups following immunization schedule with 2-month interval (A2-mixed group, A2-separate group, B2-mixed group, B2-separate group, and P2 group), the seroconversion rates for PRP antibodies reached 100% at 28 days after the second immunization (Figure 7F). In the formulation A groups (A1-mixed group, A1-separate group, A2-mixed group, and A2-separate group) and the positive control groups, the seroconversion rates for PV I antibodies reached 100% at 28 days after the first dose of immunization. In the formulation B groups, PV I-specific neutralizing antibody seropositivity reached 100% at 28 days after the second dose of immunization in the B1-mixed group, B1-separate group, and B2-separate group, and at 28 days after the third immunization in the B2-mixed group (Figure 6G and Figure 7G). The seroconversion rates for PV II antibodies reached 100% at 28 days after the first immunization in the positive control groups, while it reached 100% at 28 days after the second dose of immunization in the experimental groups (Figure 6H and Figure 7H). For PV III antibodies, the seroconversion rates reached 100% at 28 days after the second immunization in the 1-month schedule with mixed administration groups (A1-mixed group and B1-mixed group) and the 2-month schedule groups (A2-mixed group, A2-separate group, B2-mixed group, B2-separate group, and P2 group). In the immunization schedule 1 separate administration groups (A1-separate group and B1-separate group), the seroconversion rates for PV III antibodies reached 100% by 28 days following the third dose (Figure 6I and Figure 7I).

#### 3.2.2. Formulation A Induces Higher PV Type I Neutralizing Antibody Levels and Seroconversion Rates in Wistar Rats

**Figure 8:**
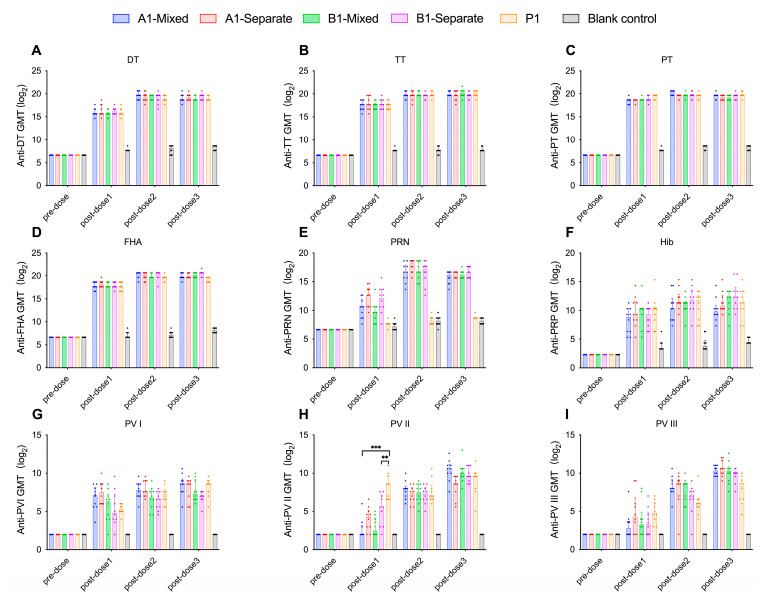
For Wistar rats immunized 1-month interval between doses, the GMTs of antibodies against DT (**A**), TT (**B**), PT (**C**), FHA (**D**), PRN (**E**), PRP (**F**), and three types of poliovirus neutralizing antibodies (**G**), (**H**) and (**I**) in each group were expressed as log2-transformed values ± 95% CIs. Statistical significance was indicated as follows: ** *p* < 0.01; *** *p* < 0.001. DT (Diphtheria Toxoid); TT (Tetanus Toxoid); PT (Pertussis Toxin); FHA (Filamentous Hemagglutinin); PRN (Pertactin); PRP (Polyribosylribitol Phosphate); PV (Poliovirus); GMT (Geometric Mean Titer).

**Figure 9:**
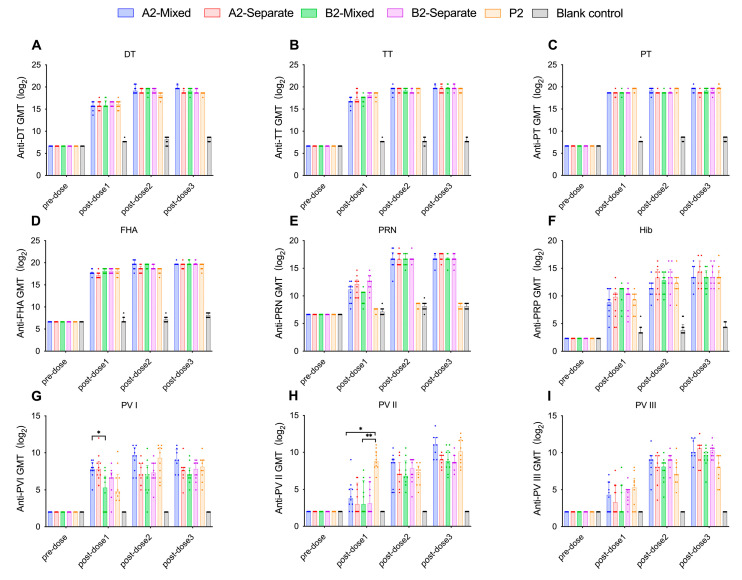
For Wistar rats immunized 2-month interval between doses, the GMTs of antibodies against DT (**A**), TT (**B**), PT (**C**), FHA (**D**), PRN (**E**), PRP (**F**), and three types of poliovirus neutralizing antibodies (**G**), (**H**) and (**I**) in each group were expressed as log2-transformed values ± 95% CIs. Statistical significance was indicated as follows: * *p* < 0.05; **p < 0.01. DT (Diphtheria Toxoid); TT (Tetanus Toxoid); PT (Pertussis Toxin); FHA (Filamentous Hemagglutinin); PRN (Pertactin); PRP (Polyribosylribitol Phosphate); PV (Poliovirus); GMT (Geometric Mean Titer).

Formulation A contains a higher concentration of IPV I antigen (30 DU) than formulation B (15 DU), while formulation B had a higher IPV II antigen content (45 DU) compared to formulation A (32 DU). After three immunizations, the PV I neutralizing antibody levels in formulation A groups were higher than those in formulation B groups. However, only after the first dose did the A2-Mixed group exhibit significantly higher PV I neutralizing antibody levels than the B2-Mixed group (*p* = 0.0493, Kruskal–Wallis H test) (Figure 9G), with no significant differences observed among other groups (*p* > 0.05, Kruskal–Wallis H test). Formulation A demonstrated relatively better performance in PV I neutralizing antibody levels and seroconversion rates, while no significant differences were observed in other components (*p* > 0.05, Kruskal–Wallis H test). PV II neutralizing antibody levels in the A1-Mixed and B1-Mixed groups were significantly lower than those in the P1 group (*p* = 0.0003, *p* = 0.0034, respectively, Kruskal–Wallis H test) (Figure 8H). Similarly, the PV II neutralizing antibody levels in the A2-mixed group and the B2-mixed group were significantly lower than those in the P2 group (*p* = 0.03, *p* = 0.0034, respectively, Kruskal–Wallis H test). No significant differences in PV III neutralizing antibody levels were observed between either formulation group and the positive control groups. Following the second and third immunizations, no significant differences in neutralizing antibody levels were observed among groups (*p* > 0.05, Kruskal–Wallis H test) (Figure 8I and Figure 9I).

#### 3.2.3. Neither Immunization Schedule nor Administration Method Significantly Affects Antigen-Specific Antibody Levels in Either Formulation

**Figure 10:**
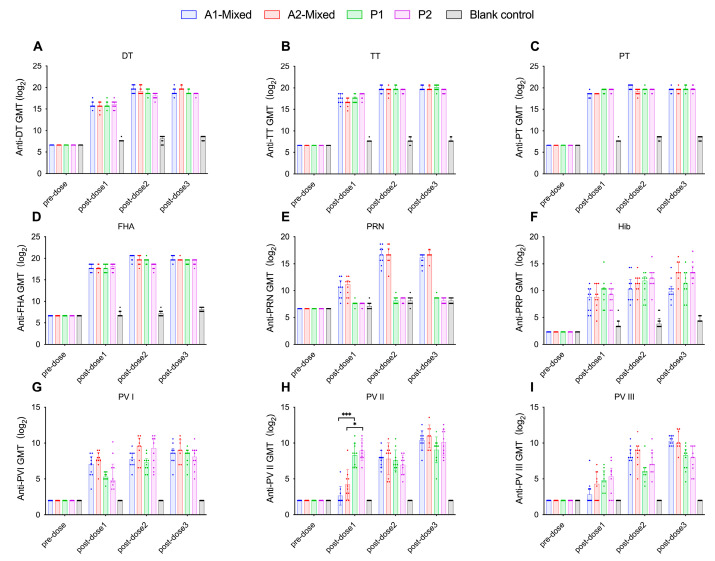
For Wistar rats immunized with formulation A, the GMTs of antibodies against DT (**A**), TT (**B**), PT (**C**), FHA (**D**), PRN (**E**), PRP (**F**), and three types of poliovirus neutralizing antibodies (**G**), (**H**) and (**I**) in each group were expressed as log2-transformed values ± 95% CIs. Statistical significance was indicated as follows: * *p* < 0.05; *** *p* < 0.001. DT (Diphtheria Toxoid); TT (Tetanus Toxoid); PT (Pertussis Toxin); FHA (Filamentous Hemagglutinin); PRN (Pertactin); PRP (Polyribosylribitol Phosphate); PV (Poliovirus); GMT (Geometric Mean Titer).

**Figure 11:**
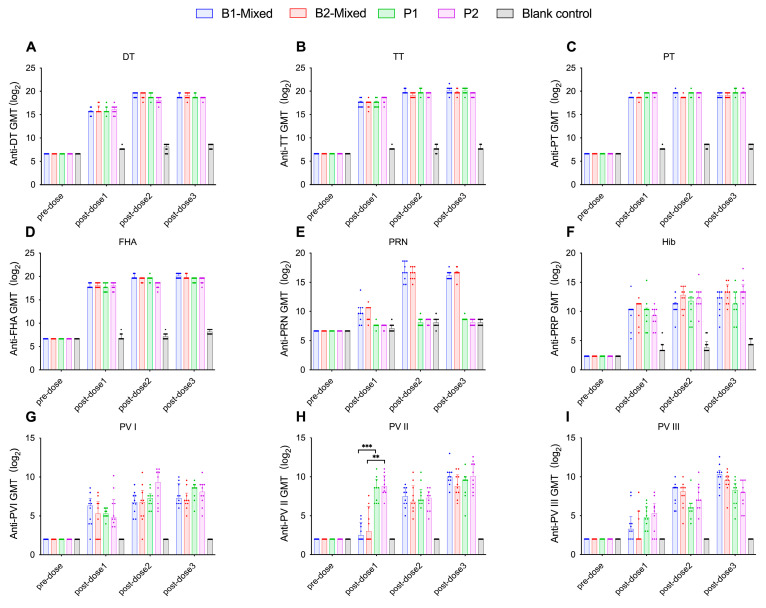
For Wistar rats immunized with formulation B, the GMTs of antibodies against DT (**A**), TT (**B**), PT (**C**), FHA (**D**), PRN (**E**), PRP (**F**), and three types of poliovirus neutralizing antibodies (**G**), (**H**) and (**I**) in each group were expressed as log2-transformed values ± 95% CIs. Statistical significance was indicated as follows: ** *p* < 0.01; *** *p* < 0.001. DT (Diphtheria Toxoid); TT (Tetanus Toxoid); PT (Pertussis Toxin); FHA (Filamentous Hemagglutinin); PRN (Pertactin); PRP (Polyribosylribitol Phosphate); PV (Poliovirus); GMT (Geometric Mean Titer).

Figure 10 illustrates the GMTs for all groups receiving formulation A under two vaccination schedules (1-month and 2-month intervals). Figure 11 presents the GMTs of groups with formulation B following the same two immunization schedules. With respect to immunization schedule, there were no significant differences in antibody levels between the immunization schedule with a 1-month interval and the schedule with a 2-month interval (*p* > 0.05, Kruskal–Wallis H test), suggesting that either schedule is appropriate for DTacP-sIPV/Hib immunization. As shown in Figure 8 and Figure 9, regarding administration method, after the first immunization, the PRN component antibody levels in the separate injection groups were slightly higher than those in the mixed injection groups, although the differences were not statistically significant (*p* > 0.05, Kruskal–Wallis H test) (Figure 8E and Figure 9E). After the second and third immunizations, no significant differences were observed in PRN component antibody levels between the mixed injection and separate injection groups (*p* > 0.05, Kruskal–Wallis H test). This suggests that separate simultaneous administration or mixed administration does not significantly impact the antibody levels of various components.

#### 3.2.4. Preliminary Safety Evaluation via Body Weight Monitoring Indicates No Detectable Impact on Growth

**Figure 12:**
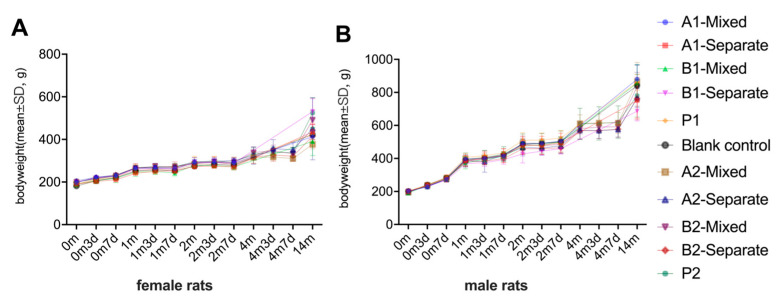
Dynamic changes in body weight in female (**A**) and male (**B**) Wistar rats. Body weights were measured prior to each immunization and on days 3 and 7 post-vaccination, with an additional measurement taken at 14 months after the first dose. “0m” indicates the time of the first dose in both immunization schedules. In the 1-month interval schedule, the second and third doses were administered at “1m” and “2m”, respectively; in the 2-month interval schedule, the second and third doses were given at “2m” and “4m”, respectively. Both female and male rats exhibited continuous weight gain throughout the study. No significant differences in body weight were observed between vaccine groups at any time point (*p* > 0.05), indicating that the candidate vaccine had no observable adverse effects on growth.

Body weight was dynamically monitored at 10 time points across all experimental groups to assess the potential impact of immunization on growth (Figure 12A,B). To standardize the timeline across immunization schedules, “0m” corresponds to the day of the first dose in both the 1-month and 2-month interval schedules. In the 1-month schedule, the second and third doses were administered at “1m” and “2m”, respectively, while in the 2-month schedule, these doses were given at “2m” and “4m”. Female and male rats were analyzed separately due to sex-related differences in body weight. All groups demonstrated consistent increases in body weight over time, with comparable growth trajectories irrespective of vaccine formulation, immunization schedule, or administration method. No significant differences in body weight were observed between vaccine groups at any time point (*p* > 0.05), suggesting that the candidate vaccine did not adversely affect growth in either sex. Repeated-measures two-way ANOVA was performed with group and time as factors. The analysis revealed a significant main effect of time (*p* < 0.0001, repeated-measures two-way ANOVA), indicating consistent growth over the course of the study. No significant differences were observed between groups (*p* > 0.05, repeated-measures two-way ANOVA), and there was no significant interaction between group and time (*p* > 0.05, repeated-measures two-way ANOVA). These findings suggest that the candidate DTacP-sIPV/Hib vaccine did not adversely affect body weight gain in rats during or after the primary immunization series.

## 4. Discussion

This study developed a novel DTacP-sIPV/Hib combination vaccine. Building on previous work on the DTacP-sIPV vaccine [19], the appropriate Hib dose was first determined using an NIH mouse model. Subsequently, experiments were conducted in Wistar rats, involving two sIPV formulations, two vaccination schedules, and two administration methods. These experiments aimed to identify the optimal immunization strategy for the candidate DTacP-sIPV/Hib vaccine.

The candidate DTacP-sIPV/Hib vaccine demonstrated a robust humoral immune response in both primary and booster immunizations, with antibody levels and seroconversion rates comparable to or better than those of the DTacP-wIPV/Hib (Pentaxim^®^), Moreover, the two Hib doses in the DTacP-sIPV/Hib vaccine induced comparable PRP antibody levels in mice. Based on antigen-sparing principles, we propose the following as an appropriate formulation for the candidate DTacP-sIPV/Hib vaccine: 12.5 Lf of DT per dose, 3.5 Lf of TT per dose, 25 μg of PT per dose, 25 μg of FHA per dose, 8 μg of PRN per dose, 10 μg of PRP per dose, and 0.725 mg of aluminum hydroxide per dose. Subsequently, we further evaluated sIPV dose, immunization schedule, and administration method of the candidate DTacP-sIPV/Hib vaccine using a Wistar rat model.

Currently, two types of sIPV formulations are commercially available in China. One formulation is produced by IMBCAMS, containing Sabin IPV antigens at 30 DU for type I, 32 DU for type II, and 45 DU for type III. Notably, the sIPV vaccine developed by IMBCAMS was the world’s first approved stand-alone Sabin strain-based inactivated poliovirus vaccine. The other formulation is produced by the Beijing Institute of Biological Products Co., Ltd. (Beijing, China) and Beijing Sinovac Biotech Co., Ltd. (Beijing, China), and contains poliovirus D antigens at 15 DU for type I, 45 DU for type II, and 45 DU for type III. Therefore, in this study, two formulations of DTacP-sIPV (Formulations A and B) were tested in Wistar rats, with differences primarily in the antigen content of sIPV I (Formulation A: 30 DU, Formulation B: 15 DU) and sIPV II (Formulation A: 32 DU, Formulation B: 45 DU). No statistically significant differences were observed in antibody titers or seroconversion rates for DT, TT, PT, FHA, PRN, PRP, PV II, or PV III between the two formulations at the same time points, regardless of the administration method (separate or mixed injection) or immunization schedule (1-month or 2-month interval). However, differences were observed in PV I antibody levels: at the same detection time points, PV I-specific neutralizing antibody titers in the Formulation A groups were consistently higher than those in the Formulation B groups under the same administration method and immunization schedule. The seroconversion rate for PV I antibodies reached 100% earlier in the Formulation A groups (after the first dose), whereas it required the second or third dose in the Formulation B groups. These results suggest that increasing the sIPV II antigen content does not significantly enhance PV II neutralizing antibody levels, while reducing the antigen content of sIPV I decreases PV I neutralizing antibody levels.

Beginning 1 January 2025, China’s National Immunization Program updated its pertussis vaccination schedule from doses at 3, 4, and 5 months (1-month intervals) with a booster at 18 months, to doses at 2, 4, and 6 months (2-month intervals), followed by boosters at 18 months and 6 years of age. Recent epidemiological studies in China have shown that 40.22% of reported pertussis cases involved individuals who had not received DTP vaccination [22]. China has also not yet implemented a maternal pertussis immunization strategy. This advancement of the first pertussis vaccine dose from 3 to 2 months of age may help reduce the disease burden in infants and young children. To align the candidate DTacP-sIPV/Hib combination vaccine with the revised schedule, two immunization schedules were tested in Wistar rats. At the same sampling points, no significant differences in antibody titers or seroconversion rates for DT, TT, PT, FHA, PRN, PV I, PV II, or PV III were observed between the two immunization schedules (1-month vs. 2-month interval), when using the same formulation and administration method. However, PRP seroconversion reached 100% after the second dose in the 2-month interval groups, whereas it required a third dose in the 1-month interval groups. Still, no statistically significant differences in PRP antibody titers or final seroconversion rates were observed between the two groups. Similarly, no significant differences in antibody titers or seroconversion rates across all components were observed between the separate and mixed administration methods with the same formulation and immunization schedule. This indicates that either separate or combined administration of DTacP-sIPV and Hib does not significantly affect antibody responses to any component.

Compared with the positive control groups (P1 and P2), no significant differences in DT, TT, PT, FHA, PRP, or PV I antibody titers or seroconversion rates were observed at corresponding time points across experimental groups, regardless of formulation, administration method (separate or mixed), or immunization schedule (1-month or 2-month interval). As Pentaxim^®^ lacks the PRN antigen, it does not induce PRN antibodies in the positive control groups. After the first dose, PV II and PV III antibody titers were significantly higher in the positive control groups than in the experimental groups (both *p* < 0.05). However, no significant differences were observed after the second and third doses (both *p* > 0.05). Both clinical and preclinical studies of sIPV-based combination vaccines have revealed immunogenicity differences between sIPV and wIPV. Most studies report that sIPV induces higher neutralizing antibody titers against poliovirus type I, slightly lower titers against type II, and comparable titers against type III compared to wIPV [19,23,24,25]. Clinical studies of combination vaccines containing sIPV have generally shown no significant differences in neutralizing antibody titers against any poliovirus serotype between sIPV and wIPV, following completion of the three-dose primary immunization [26,27]. Similarly, preclinical studies have found equivalent titers across all poliovirus types following primary immunization with either IPV type [19,24].

## 5. Conclusions

The candidate DTacP-sIPV/Hib vaccine elicited strong humoral immune responses in both NIH mice and Wistar rats. Following three primary immunizations, antibody levels against all antigen components were comparable to those in the positive control group (Pentaxim^®^). No statistically significant differences in antibody responses were observed between the 1-month and 2-month immunization schedules, or between separate and mixed administration of DTacP-sIPV and Hib. Modifying the sIPV I and II antigen contents (from 30 to 15 DU and 32 to 45 DU, respectively) did not significantly affect antibody levels against DT, TT, PT, FHA, PRN, PRP, PV II, or PV III in rats. However, it led to reduced PV I antibody titers and seroconversion rates. Therefore, we recommend the following formulation as the optimal dose for the DTacP-sIPV/Hib candidate vaccine: DT 12.5 Lf, TT 3.5 Lf, PT 25 μg, FHA 25 μg, PRN 8 μg, PRP 10 μg, sIPV I 30 DU, sIPV II 32 DU, sIPV III 45 DU, and aluminum hydroxide 0.725 mg per dose. Additionally, reconstituting and mixing Hib with DTacP-sIPV before injection and administering three doses primary immunization using either a 1-month or 2-month interval represents a feasible immunization strategy. Future studies will assess the immune persistence and booster effects of the candidate DTacP-sIPV/Hib vaccine in rats and further evaluate its immunogenicity in rhesus macaques to support upcoming clinical trials.

## Figures and Tables

**Table 1:** Composition of vaccines used in NIH mouse.

Vaccine	DTacP-sIPV/Hib (Low-Dose)	DTacP-sIPV/Hib (High-Dose)	DTacP-wIPV/Hib (Pentaxim^®^)
PT (μg/dose)	25	25	25
FHA (μg/dose)	25	25	25
PRN (μg/dose)	8	8	-
DT (Lf/dose)	12.5	12.5	15
TT (Lf/dose)	3.5	3.5	5
IPV I (DU/dose)	30	30	40
IPV II (DU/dose)	32	32	8
IPV III (DU/dose)	45	45	32
PRP (μg/dose)	10	15	10
Al(OH)_3_ (mg/dose)	0.725	0.725	0.91

PT (Pertussis Toxin); FHA (Filamentous Hemagglutinin); PRN (Pertactin); DT (Diphtheria Toxoid); TT (Tetanus Toxoid); PRP (Polyribosylribitol Phosphate); IPV (Inactivated Poliovirus Vaccine).

**Table 2:** Composition of vaccines used in Wistar rats.

Vaccine	DTacP-sIPV/Hib Formulation A	DTacP-sIPV/Hib Formulation B	DTacP-wIPV/Hib (Pentaxim^®^)	Normal Saline
PT (μg/dose)	25	25	25	/
FHA (μg/dose)	25	25	25	/
PRN (μg/dose)	8	8	/	/
DT (Lf/dose)	12.5	12.5	15	/
TT (Lf/dose)	3.5	3.5	5	/
PRP (μg/dose)	10	10	10	/
IPV I (DU/dose)	30	15	40	/
IPV II (DU/dose)	32	45	8	/
IPV III (DU/dose)	45	45	32	/
Al(OH)_3_ (mg/dose)	0.725	0.725	0.91	/

PT (Pertussis Toxin); FHA (Filamentous Hemagglutinin); PRN (Pertactin); DT (Diphtheria Toxoid); TT (Tetanus Toxoid); PRP (Polyribosylribitol Phosphate); IPV (Inactivated Poliovirus Vaccine).

**Table 3:** Wistar rats grouping and vaccination strategy.

Group	Name	Vaccine	Immunization Schedule	Administration
Group 1	A1-Mixed	DTacP-sIPV/Hib Formulation A	1-month interval	Mixed Injection
Group 2	A1-Separate	DTacP-sIPV/Hib Formulation A	1-month interval	Separate Injection
Group 3	B1-Mixed	DTacP-sIPV/Hib Formulation B	1-month interval	Mixed Injection
Group 4	B1-Separate	DTacP-sIPV/Hib Formulation B	1-month interval	Separate Injection
Group 5	P1	DTacP-wIPV/Hib (Pentaxim^®^)	1-month interval	Mixed Injection
Group 6	Blank control	Normal Saline	1-month interval	/
Group 7	A2-Mixed	DTacP-sIPV/Hib Formulation A	2-month interval	Mixed Injection
Group 8	A2-Separate	DTacP-sIPV/Hib Formulation A	2-month interval	Separate Injection
Group 9	B1-Mixed	DTacP-sIPV/Hib Formulation B	2-month interval	Mixed Injection
Group 10	B1-Separate	DTacP-sIPV/Hib Formulation B	2-month interval	Separate Injection
Group 11	P2	DTacP-wIPV/Hib (Pentaxim^®^)	2-month interval	Mixed Injection

## Data Availability

The data generated and analyzed during the current study are available from the corresponding author upon reasonable request.

## Abbreviations

The following abbreviations are used in this manuscript:

DTaP	Diphtheria, Tetanus, and acellular Pertussis vaccine
DTP	Diphtheria, Tetanus, and Pertussis vaccine
aP	acellular Pertussis vaccine
acP	acellular component Pertussis vaccine
Hib	*Haemophilus influenzae* type b
IPV	Inactivated Poliovirus Vaccine
sIPV	Sabin strain Inactivated Poliovirus Vaccine
wIPV	Wild strain Inactivated Poliovirus Vaccine (Salk strain)
DTacP-sIPV/Hib	Diphtheria, Tetanus, acellular component Pertussis, Sabin strain Inactivated Poliovirus and *Haemophilus influenzae* type b combination vaccine
DTacP-wIPV/Hib	Diphtheria, Tetanus, acellular component Pertussis, wild strain Inactivated Poliovirus and *Haemophilus influenzae* type b combination vaccine
HBV	Hepatitis B Virus vaccine
NIP	National Immunization Program
IMBCAMS	Institute of Medical Biology, Chinese Academy of Medical Sciences
NIH	National Institutes of Health (mouse strain)
SPF	Specific Pathogen-Free
IM	Intramuscular
IP	Intraperitoneal
ELISA	Enzyme-Linked Immunosorbent Assay
ANOVA	Analysis of variance
GMT	Geometric Mean Titer
GMC	Geometric mean concentration
SD	Standard Deviation
CIs	Confidence intervals
IQR	Interquartile range
DT	Diphtheria Toxoid
TT	Tetanus Toxoid
PT	Pertussis Toxin
FHA	Filamentous Hemagglutinin
PRN	Pertactin
PRP	Polyribosylribitol Phosphate
OD	Optical density
